# Effects of dietary *Astragalus membranaceus* and *Codonopsis pilosula* extracts on growth performance, antioxidant capacity, immune status, and intestinal health in broilers

**DOI:** 10.3389/fvets.2023.1302801

**Published:** 2023-12-07

**Authors:** Shun Liu, Gengsheng Xiao, Qi Wang, Jinpeng Tian, Xin Feng, Qingyang Zhang, Li Gong

**Affiliations:** ^1^Guangdong Provincial Key Laboratory of Animal Molecular Design and Precise Breeding, School of Life Science and Engineering, Foshan University, Foshan, Guangdong, China; ^2^Key Laboratory of Animal Molecular Nutrition of Education of Ministry, College of Animal Science, Zhejiang University, Hangzhou, China

**Keywords:** Chinese herbal medicine extract, *Astragalus membranaceus*, *Codonopsis pilosula*, growth performance, antioxidation, immune response, intestinal barrier function, gut microbiota

## Abstract

The objective of this study was to examine the effects of dietary Chinese herbal medicine (CHM) consisting of *Astragalus membranaceus (Fisch.) Bunge* (AMT) and *Codonopsis pilosula (Franch.) Nannf* (CPO) extracts on growth performance, antioxidant capacity, immune status, and intestinal health of broiler chickens. Two groups were formed, each consisting of six replicates of 12 one-day-old healthy male 817 white feather broilers. Broilers were fed either a basal diet (CON group) or a basal diet supplemented with 500 mg/kg CHM. The trial lasted 50 days. The results showed that CHM supplementation resulted in enhanced feed efficiency and antioxidant capacity in both the serum and liver, while it reduced uric acid and endotoxin levels, as well as diamine oxidase activity (*p* < 0.05). Additionally, CHM treatment increased the height of jejunum villi and upregulated *Claudin-1* expression in the jejunal mucosa accompanied by an increase in the mRNA levels of interleukin-6 (IL-6), interferon-γ (IFN-γ), interferon-β (IFN-β), tumor necrosis factor-α (TNF-α), and anti-inflammatory cytokine interleukin-10 (IL-10) (*p* < 0.05). The presence of dietary CHM caused an increase in the proportions of *Bacteroidetes* and unclassified *Bacteroidales* but led to a decrease in those of *Firmicutes* and *Alistipes* (*p* < 0.05). The composition of the jejunal mucosa microbiota was correlated with the feed conversion ratio, serum metabolites, and gene expression based on Spearman correlation analysis. The findings indicated that the consumption of dietary CHM improved the utilization of feed, increased the mRNA expression of pro-inflammatory cytokines in the jejunal mucosa, and decreased the endotoxin level and activities of diamine oxidase and lactate dehydrogenase in the serum, which could potentially be linked to changes in the gut microbiota of broiler chickens.

## 1 Introduction

In recent years, chicken meat has been in high demand as a source of protein. Consequently, commercial chicken farms prioritize faster growth and higher feed efficiency as their primary objectives. However, attaining these objectives requires ensuring the health of the chickens, good digestion and absorption, and protection against pathogens. Owing to the exhaustive ban on antibiotics in feed, broiler chickens have become increasingly vulnerable to illnesses, resulting in diminished performance as they face the threat of contagious agents, stressors, and nutritional insufficiencies (1, 2). Hence, many researchers are focusing on finding effective solutions to address or mitigate this issue.

Chinese herbal medicines (CHM) and their extracts have been utilized as adjuvants to mitigate the risk of disease and enhance the growth performance of livestock (3–5). The profusion of herbal remedies, *Astragalus membranaceus (Fisch.) Bunge* (AMT) and *Codonopsis pilosula (Franch.) Nannf* (CPO) has been used in traditional oriental medicine. AMT, one of the most widely used tonic herbs in many Asian regions, has been reported to possess components that demonstrate various biological effects, including antioxidative, anti-inflammatory, and antiviral properties (6–8). AMT has been reported to scavenge oxygen free radicals, making it an important plant in preventing mucosal injury in the intestine, liver, and plasma (9). CPO is known for its ability to enhance spleen function, promote liver health, and exhibit anti-tumor, antioxidant, and antibacterial effects (10–12). Studies have also shown that AMT and CPO have immunomodulatory effect, improve immune defense function of broiler chickens and mice, and have nourishing effects and can be used as immune adjuvants (13, 14). These two plants are often used in tandem to combat multiple of diseases because their synergistic effects are believed to promote lipid oxidation, protect against illnesses, and ameliorate inflammation (15, 16).

Recently, researchers found that the combination of CPO and AMT could re-establish the immune balance of the intestinal flora and alleviate colonic mucosal injury in mice with colitis (16). Synergistic extracts of AMT and CPO showed antioxidant activity in weaned piglets and influenced the relative proportion of Firmicutes and Bacteroidetes in the gastrointestinal tract (17). However, to the best of our knowledge, the combined effects of AMT and CPO extracts in broiler chickens have not yet been thoroughly studied. Therefore, the aim of this study was to explore the effects of adding CHM, by considering the example of AMT and CPO extracts, to the diet on the growth performance, immune response, antioxidant capacity, and intestinal health of broiler chickens.

## 2 Materials and methods

### 2.1 Experimental design, birds, diets, and management

A total of 144 one-day-old male 817 white feather broilers (Nanhai little white chicken) with similar initial weights were purchased from Muyuan Foods Co., Ltd., (Guangzhou, China). The broilers were allocated to two groups, with six replicates per group and 12 chicks per replicate. Birds were fed with either a basal diet or a basal diet supplemented with 500 mg/kg CHM. The determination of the CHM dosage was based on a preliminary experiment conducted by the research group members, taking into account the dosage of extracts mentioned in our previous study (18). The CHM product was purchased from Guangdong Huakang Biopharmaceutical Co., LTD (Guangzhou, China), which mainly consists of a mixture of extracts of AMT and CPO. The main component of this product is polysaccharide. The diet was developed based on the nutrient needs of broiler chickens (Table 1), ensuring that all essential nutrients were provided in amounts that met or surpassed the requirements set for chickens (NRC, 1994). In this study, all the chicks were kept in a controlled environment with specific dark-and-light cycles. All chicks were subjected to the same light cycle arrangement (24 L:0 D) 24 h before the experiment. From the second day of age, the brightness gradually decreased and reached 18 L:6 D on the seventh day. From the eighth day onwards, the light cycle was maintained at 16 L:8 D until the end of the experiment. Furthermore, the chicks were provided with unrestricted access to both water and feed throughout the entire duration of the trial.

**Table 1:** Composition and nutrient content of experimental diets.

Diets	Starter (1–20 days)	Finisher (21–50 days)
Ingredients (g/kg)		
Corn	58.75	57.66
Soybean meal (43% CP)	27.70	27.21
Corn gluten meal (60% CP)	5.00	5.00
Limestone	1.43	1.22
Calcium hydrogen phosphate (16.5%)	1.10	1.04
L-lysine Sulfate (70%)	0.66	0.39
DL-methionine (98.5%)	0.33	0.21
NaCl	0.28	0.32
L-Threonine	0.16	0.06
Peanut meal	3.00	0
Choline chloride (50%)	0.08	0.08
Premix ^1^	0.35	0.35
Phytase (20,000 IU)	0.01	0.01
Sodium humate	0	0.15
Lard	1.15	6.30
Total (%)	100	100
Nutrient content ^2^		
ME (Kcal/kg)	2941.61	3231.28
CP (g/kg)	21.92	19.61
CEE (g/kg)	3.65	8.87
*CF* (g/kg)	2.51	2.33
Ca (g/kg)	0.90	0.80
Total P (%)	0.57	0.54
Lys (%)	1.48	1.21
Met (%)	0.64	0.52
Cys (%)	0.30	0.27
Met+Cys (%)	0.94	0.78
L-Threonine	0.92	0.79

^1^ Premix is provided for each kilogram of diet: vitamin A, 12,000 IU; vitamin D3, 3,000 IU; vitamin E, 10 IU; vitamin K3, 2 mg; vitamin B1, 1 mg; vitamin B2, 3 mg; vitamin B6, 2 mg; vitamin B12, 0.01 mg; niacin, 20 mg; calcium pantothenate, 4 mg; biotin, 0.05 mg; folic acid, 0.5 mg; Mn, 100 mg; Fe, 100 mg; Zn, 80 mg; Cu, 20 mg; I, 3 mg; and Se, 0.5 mg.^2^ Nutrient levels were calculated values.ME, metabolizable energy; CP, crude protein; CEE, crude ether extract; *CF*, crude fiber.

### 2.2 Sample collection

On the 50th day, after an eight-hour fasting period, measurements were made of the body weight (BW) and the amount of feed remaining in each replicate. Afterwards, the growth performance parameters for each replicate were computed, which included the mean average daily feed intake (ADFI), mean average daily gain (ADG), and the feed conversion ratio (FCR). FCR = feed intake (g) / weight gain (g). Additionally, any instances of mortality throughout the entire 50-day duration of the experiment were taken into consideration. One chicken was selected from each replicate after weighting at d 50 for blood and tissue samples collection. Following the extraction of around 4 mL of blood from the brachial vein, it was subsequently centrifuged at a temperature of 4°C for 15 min with a force of 3,000 g. After centrifugation, serum was stored at −80°C for further analysis. Following blood collection, chickens were sacrificed through exsanguination. Liver tissue samples were collected uniformly from the left side of each chicken, immediately placed in liquid nitrogen, and subsequently stored at −80°C to facilitate determination of antioxidant capacity. Furthermore, the jejunum, jejunal mucosa, and the contents of the cecum were systematically collected and preserved using different techniques. Both the jejunal mucosa and jejunal tissue samples were obtained from the middle section of the intestinal segment. The jejunal mucosa was collected by scraping the jejunum using a scalpel. Additionally, the scraped jejunal tissue, measuring approximately 3–4 cm in length, was preserved for further analysis. Histomorphological analyses were conducted on sections of scraped jejunal tissue that were preserved in formaldehyde.

### 2.3 Serum biomarkers measurement

Serum biomarkers were tested using detection kits from the Institute of Bioengineering, Nanjing Jiancheng (Nanjing, China). These markers include alanine aminotransferase, alkaline phosphatase, total cholesterol, albumin, low-density lipoprotein cholesterol, triglycerides, high-density lipoprotein cholesterol (HDL-C), lactate dehydrogenase (LDH), and uric acid (UA). Additionally, diamine oxidase (DAO) and endotoxin concentrations were measured a DAO assay kit and an endotoxin detection kit, respectively. Interleukin 2 (IL-2), interleukin-10 (IL-10), interleukin-6 (IL-6), tumor necrosis factor-α (TNF-α), secretory immunoglobulin A (sIgA) and transforming growth factor-β (TGF-β) were measured in each group with chicken specific enzyme-linked immunosorbent assay (ELISA) kits (Nanjing Jiancheng Biomedicine, China). All determination procedures were conducted following the instructions given by the manufacturer (Nanjing Jiancheng Biomedicine, China).

### 2.4 Serum and liver antioxidant capacity

The antioxidant levels in serum and livers were assessed with kits to measure various indicators, including total superoxide dismutase (T-SOD), malondialdehyde (MDA), catalase (CAT), total antioxidant capacity (T-AOC), and glutathione peroxidase (GSH-Px). The kits were purchased from Nanjing Jiancheng BioEngineering, and the determination procedure was conducted following the kits’ instructions. Before homogenizing with nine times normal saline, the liver samples were thawed on ice, maintaining a ratio of 1 gram of tissue to 9 milli-liters of saline. The website of Nanjing Jiancheng company has a technical support section that offers details about technical approaches.^1^ After centrifugation, the supernatant from liver homogenate was obtained for determining and analyzing the index.

### 2.5 Intestinal tissue morphology

Morphology is an accepted method for studying the change of tissue structure, such as livers (19), hearts (20), and jejunum (21). In this study, tissue specimens from the jejunum were collected and preserved in a formaldehyde solution for further analysis. Afterwards, the samples were immersed in paraffin and sliced into segments. The serial sections that were affixed onto a slide, which was then treated with hematoxylin and eosin staining. Six complete crypto-villi units were selected from each sample for analysis. Measurements of the small intestine villi and crypts were performed using the high-definition LEICA imaging system (version DFC290, Heilbrugger, Switzerland). To assess the morphology of the tissue, measurements of villus height and crypt depth were taken. The measurement of villi height starts from the villi tip and ends at the junction of the villi recess, while crypt depth refers to the depth of the invagination between neighboring villi. Calculate the villus/crypt ratio by dividing the villi height by the crypt depth. This ratio provides insight into the structural integrity of the intestinal tissue.

### 2.6 Quantitative PCR

We used TransZol reagent (TransGen Biotech, Beijing, China) to extract RNA from jejunal mucosa and with the NanoDrop 2000C Ultramicrospectrophotometer (Thermo, Shanghai, China) to measure the quality of the RNA (OD 260/280). RNA was reverse-transcribed by RT EasyTMII Kit, and cDNA was synthesized. A specific primer sequence was combined with cDNA, and real-time quantitative PCR was conducted to analyze cDNA gene expression. The primers were created with the assistance of the NCBI primer tool and their specificities were confirmed. Qingke Biological Co., Ltd. synthesized the primers utilized in the research, and all pairs of primers exhibited an amplification efficiency of approximately 100% (Table 2). The PCR amplification system contained a total volume of 20 μL, and the cycling conditions consisted of 30 s at 95°C, followed by 10 s at 95°C and 30 s at 60°C for 40 cycles. To determine the relative gene expression, real-time quantitative PCR was used using the 2^−∆∆Ct^ method (20, 22).

**Table 2:** The primer sequence of the gene.

Gene	GenBank	Sequence (5′–3′)	TM °C
*β-actin*	NM_205518.2	F: CATTGTCCACCGCAAATGCT R: AAGCCATGCCAATCTCGTCT	57.2
*IL-6*	NM_204628.2	F: CAGGACGAGATGTGCAAGAA R: TAGCACAGAGACTCGACGTT	56.6
*IL-10*	NM_001004414.4	F: CGCTGTCACCGCTTCTTCA R: CTTTGTTCTCATCCATCTTCTC	57.0
*IL-1β*	XM_046931582.1	F: CGACATCAACCAGAAGTGCTT R: GTCCAGGCGGTAGAAGATGA	56.8
*IL-22*	NM_001199614.1	F: GCCCTACATCAGGAATCGCA R: TCTGAGAGCCTGGCCATTTC	57.8
*IL-17A*	NM_204460.2	F: GAAGGTGATACGGCCAGGAC R: TGGGTTAGGCATCCAGCATC	56.8
*IFN-β*	NM_001024836.2	F: TGCAACCATCTTCGTCACCA R: GGAGGTGGAGCCGTATTCT	56.68
*IFN-γ*	NM_205149.2	F: ACACTGACAAGTCAAAGCCGC R: AGTCGTTCATCGGGAGCTTG	58.6
*TNF-α*	XM_046927265.1	F: TGTGTATGTGCAGCAACCCGTAGT R: GGCATTGCAATTTGGACAGAAGT	57.8
*MUC-2*	XM_040673077.2	F: TTCATGATGCCTGCTCTTGTG	58.0
		R: CCTGAGCCTTGGTACATTCTTGT	
*ZO-1*	XM_046925214.1	F: CTTCAGGTGTTTCTCTTCCTCCTC	56.6
		R: CTGTGGTTTCATGGCTGGATC	
*Claudin-1*	NM_001013611.2	F: GGTATGGCAACAGAGTGGCT R: CAGCCAATGAAGAGGGCTGA	57.0
*Occludin*	XM_046904540.1	F: GATGGACAGCATCAACGACC R: CATGCGCTTGATGTGGAAGA	58.0

IL-6, interleukin-6; IL-10, interleukin-10; IL-1β, interleukin-1β; IL-22, interleukin-22; IL-17A, interleukin-17A; IFN-γ, interferon-γ; IFN-β, interferon-β; TNF-α, tumor necrosis factor-α; MUC-2, Mucin-2; ZO-1, Zonula occludens-1.

### 2.7 16S sequencing and cecal microbiota analysis

The 16 s rRNA sequencing of feces was entrusted to Lianchuan Biotechnology Co., LTD (Hangzhou, China). The hexadecyltrimethylammonium bromide (CTAB) method was used to extract genomic DNA from the contents of the cecum. The concentration of DNA was determined and the quality of the DNA extraction was evaluated using agarose gel electrophoresis, employing an ultraviolet spectrophotometer. DNA obtained from the samples was used to amplify the 16S rRNA V3-V4 region. PCR products were purified using AM-Pure XT beads from Beckman Coulter Genomics in Danvers, MA, USA. Subsequently, the quantification was performed using Qubit from Invitrogen in the United States. The refined PCR samples were evaluated using an Agilent 2,100 Bioanalyzer (Agilent, USA) and the Illumina Library Quantification Kit from Kapa Biosciences (Woburn, MA, USA). The samples obtained through sequencing were divided based on barcode information for the double-ended data. Data was spliced and filtered after the joint and barcode sequences were removed. Afterwards, dada 2 was used in conjunction with qiime DADA 2 denoise-paired to carry out length filtering and denoising, which led to the detection of ASV (feature) sequences and the generation of an ASV (feature) abundance chart. Singletons ASV were then removed. The acquired ASV (characteristic) sequences and ASV (characteristic) abundance table were utilized for the analysis of alpha diversity and beta diversity. The alpha diversity analysis assesses domestic diversity using six indexes: observed_species, shannon, simpson, chao1, goods_coverage, and pielou_e. Additionally, four types of distances (unweighted_unifrac, weighted_unifrac, jaccard, bray_curtis) were calculated to evaluate the diversity between habitats (samples/groups). The characterization of microorganismal features differentiating the fecal microbiota was performed using the linear discriminant analysis (LDA) effect size (LEfSe) method.^2^ We analyzed the Spearman’s correlations using the heatmap function from the R package (version 3.6.3). Spearman correlation methodology can be the website,^3^ and use OmicStudio tools on^4^ perform clustering correlation heat map with symbols (23).

### 2.8 Statistical analysis

All data was analyzed using independent samples *t*-test in SPSS version 23.0 software (SPSS, Inc., Chicago, IL, USA). The data were expressed as mean ± standard deviation (SD). Values at *p* < 0.05 were statistically significant and values at 0.05 < *p* < 0.10 are trending.

## 3 Results

### 3.1 Growth performance

Data on growth performance are shown in Table 3. Compared with those in the CON group, the BW and ADG were higher in the CHM group; however, there were no significant differences observed among BW, ADG, and ADFI between the two groups from day 1 to day 50. However, FCR of the CHM group was lower than that of the CON group (*p* = 0.02).

**Table 3:** Effects of CHM on growth performance of broilers.

Items	CON	CHM	*p*-value
1 d BW, g	36.20 ± 0.01	36.2 ± 0.01	1.00
50 d BW, g	2015.00 ± 62.65	2036.67 ± 120.97	0.80
1–50 d			
ADG, g/d	40.38 ± 1.28	40.83 ± 2.47	0.80
ADFI, g/d	78.34 ± 3.52	74.31 ± 2.64	0.08
FCR	1.94 ± 0.06	1.82 ± 0.02	0.02

BW, body weight; ADG, average daily gain; ADFI, average daily feed intake; FCR, feed conversion ratio; CON, control; CHM, Chinese herbal medicine.

### 3.2 Serum physiological and biochemical indexes

The effects of CHM on the physiological serum parameters of broilers are presented in Table 4. Compared with the CON group, the CHM group showed a significant increase in total cholesterol (*p* = 0.03) and HDL-C (*p* = 0.01) serum levels, while exhibiting a decrease in UA level (*p* = 0.01) and LDH activity (*p* = 0.03). Aspartate aminotransferase level (*p* = 0.05) was higher in the CHM group than in the CON group. In addition, the CHM group exhibited a significant decrease in DAO activity (*p* = 0.01) and endotoxin concentration (*p* = 0.04). Serum levels of triglycerides, low-density lipoprotein cholesterol, albumin, alkaline phosphatase, and alanine aminotransferase were not significantly affected by the experimental treatments.

**Table 4:** Effects of CHM on serum physiological and biochemical indexes of broilers.

Items	CON	CHM	*p*-value
Triglycerides, mmol/L	0.75 ± 0.19	0.87 ± 0.13	0.20
Total cholesterol, mmol/L	4.26 ± 0.67	5.30 ± 0.75	0.03
HDL-C, mmol/L	3.62 ± 1.51	5.76 ± 0.87	0.01
Low-density lipoprotein cholesterol, mmol/L	3.43 ± 0.86	3.02 ± 0.57	0.36
Albumin, g/L	20.87 ± 4.03	22.17 ± 2.56	0.52
UA, μmol/L	353.52 ± 72.75	250.44 ± 26.51	0.01
LDH, U/L	6072.58 ± 257.85	5529.26 ± 437.67	0.03
Alkaline phosphatase, mg/L	155.56 ± 29.88	154.94 ± 53.75	0.98
Aspartate aminotransferase, U/L	15.36 ± 3.88	24.68 ± 9.77	0.05
Alanine aminotransferase, U/L	21.12 ± 12.44	14.30 ± 6.60	0.26
DAO, U/L	18.13 ± 5.28	7.88 ± 1.74	0.01
Endotoxin, U/L	1.43 ± 0.50	0.83 ± 0.42	0.04

HDL-C, high-density lipoprotein cholesterol; UA, uric acid; LDH, lactate dehydrogenase; DAO, diamine oxidase; CON, control; CHM, Chinese herbal medicine.

### 3.3 Immune response

As shown in Table 5, serum levels of immune factors were determined using enzyme-linked immunosorbent assay (ELISA) kits, whereas mRNA levels of immune factors in the jejunal mucosa of broilers were quantified using quantitative polymerase chain reaction (qPCR). Compared with the CON group, the CHM group exhibited decreased levels of IL-10 (*p* = 0.08) in the serum. Moreover, birds in CHM group had increased mRNA expression levels of pro-inflammatory cytokines IL-6 (*p* = 0.02), interferon-γ (IFN-γ) (*p* = 0.01), interferon-β (IFN-β) (*p* = 0.01) and TNF-α (*p* = 0.01), as well as anti-inflammatory cytokine IL-10 (*p* = 0.01) in the jejunal mucosal tissue compared to the birds in the CON group.

**Table 5:** Effects of CHM on the expression levels of immune factors in the serum and jejunal mucosa of broilers.

Items	CON	CHM	*p*-value
Serum			
IL-2, ng/L	22.712 ± 5.30	27.47 ± 6.03	0.18
IL-6, ng/L	94.03 ± 15.99	92.18 ± 20.90	0.87
TNF-α, ng/L	193.65 ± 14.71	183.97 ± 13.68	0.25
TGF-β, ng/L	2652.14 ± 242.39	2354.87 ± 340.84	0.11
IL-10, ng/L	4.80 ± 1.66	3.35 ± 0.71	0.08
sIgA, ng/L	170.80 ± 16.67	188.50 ± 36.93	0.30
Jejunal mucosa mRNA abundance			
IL-6	1.00 ± 0.02	2.38 ± 0.83	0.02
IL-1β	1.00 ± 0.03	1.42 ± 0.69	0.29
IL-22	1.00 ± 0.01	1.12 ± 0.65	0.72
IL-17A	1.00 ± 0.01	1.66 ± 1.06	0.26
IFN-γ	1.00 ± 0.01	2.16 ± 0.31	0.01
TNF-α	1.03 ± 0.04	2.57 ± 0.62	0.01
IL-10	1.00 ± 0.01	3.56 ± 0.90	0.01
IFN-β	1.00 ± 0.02	1.83 ± 0.26	0.01

IL-2, interleukin-2; IL-6, interleukin-6; IL-10, interleukin-10; IL-22, interleukin-22; IL-17A, interleukin-17A; IFN-γ, interferon-γ; IFN-β, interferon-β; TNF-α, tumor necrosis factor-α; TGF-β, transforming growth factor-β; sIgA, secretory immunoglobulin A; CON, control; CHM, Chinese herbal medicine.

### 3.4 Serum and liver antioxidant capacity

Compared with the CON group (Table 6), the CHM group exhibited a significant increase in serum antioxidant parameters, specifically T-AOC (*p* = 0.01), T-SOD (*p* = 0.02) and GSH-Px (*p* = 0.02) content. In addition, the CHM group showed significantly increased T-AOC (*p* = 0.01), T-SOD (*p* = 0.01), CAT (*p* = 0.04) and GSH-Px activity (*p* = 0.01) in the liver tissues compared to the CON group.

**Table 6:** Effects of CHM on serum and hepatic antioxidant capacity of broilers.

Items	CON	CHM	*p*-value
Serum			
MDA, nmol/mL	3.52 ± 1.09	2.98 ± 9.62	0.26
CAT, U/mL	4.03 ± 0.88	5.02 ± 1.60	0.21
T-AOC, U/mL	3.86 ± 1.08	7.57 ± 2.13	0.01
T-SOD, U/mL	45.03 ± 6.8	54.47 ± 4.15	0.02
GSH-Px, μmol/L	1249.27 ± 273.88	1639.38 ± 175.37	0.02
Liver			
MDA, nmol/mgprot	5.13 ± 0.80	4.40 ± 0.68	0.12
T-AOC, U/mgprot	5.26 ± 0.34	6.71 ± 0.78	0.01
CAT, U/mgprot	18.14 ± 4.75	24.71 ± 4.72	0.04
T-SOD, U/mgprot	265.40 ± 30.89	339.08 ± 40.55	0.01
GSH-Px, μmol/mgprot	63.95 ± 7.31	93.64 ± 18.69	0.01

MDA, malondialdehyde; CAT, catalase; T-AOC, total antioxidant capacity; T-SOD, total superoxide dismutase; GSH-Px, glutathione peroxidase; CON, control; CHM, Chinese herbal medicine.

### 3.5 Intestinal physical barrier function

As shown in Table 7, CHM supplementation significantly increased the height of the jejunal villi (*p* = 0.01) and upregulated the mRNA expression level of Claudin-1 (*p* = 0.01) in the jejunal mucosa compared to those in the CON group.

**Table 7:** Effects of CHM on jejunal morphology and the mRNA expression of mucosa tight junction protein.

Items	CON	CHM	*p*-value
50 d villus height, μm	1066.44 ± 49.25	1237.66 ± 88.57	0.01
50 d crypt depth, μm	112.51 ± 11.99	113.83 ± 9.62	0.84
50 d V/C	9.71 ± 0.95	10.92 ± 1.00	0.06
ZO-1	0.99 ± 0.01	1.06 ± 0.44	0.77
MUC-2	1.01 ± 0.02	1.44 ± 0.91	0.38
Claudin-1	1.00 ± 0.03	2.28 ± 0.44	0.01
Occludin	1.01 ± 0.05	0.84 ± 0.39	0.41

V/C, villus height / crypt depth; ZO-1, Zonula occludens-1; MUC-2, Mucin-2; CON, control; CHM, Chinese herbal medicine.

### 3.6 Gut microbiota

**Figure 1:**
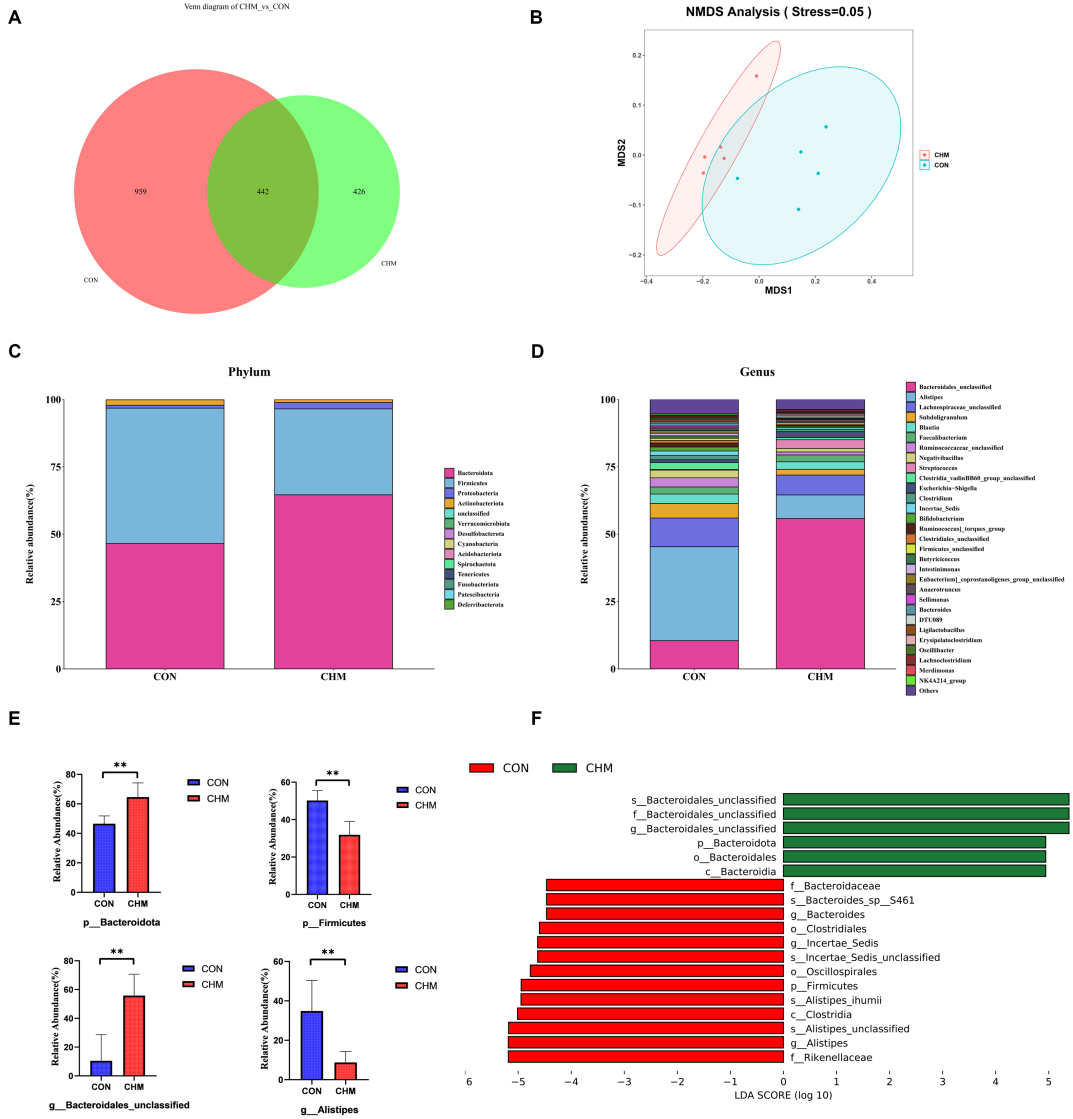
Relative abundance of the cecal microbiota. **(A)** Venn diagram of OTUs; number; **(B)** NMDS using Bray-curtis distance; **(C)** Phylum-level taxonomic composition of the cecal microbiota; **(D)** Genus-level taxonomic composition and relative abundance of the cecal microbiota; **(E)** The relative abundance of Phylum Firmicutes and Bacteroidetes and genus *Bacteroidales_unclassified* and *Alistipes*. **(F)** Lefse identified the most differential genera between the CON group and the CHM group. CON, control; CHM, Chinese herbal medicine. Significant differences are denoted by asterisks (*p < 0.05; **p < 0.01).

Figure 1 revealed that CHM supplementation affected broiler cecal microbiota. According to the Venn diagram, the overall number of operational taxonomic units (OTUs) was 1,827, with 442 OTUs common to both groups (Figure 1A). Notably, 426 OTUs were found to be unique to the CHM group. To depict the microbiome space of various groups, we performed non-metric multidimensional scaling (NMDS) using weighted UniFrac and Bray–Curtis distances. The composition of the gut microbiome differed between the two groups (Figure 1B). In contrast to that in the CON group, the inclusion of CHM significantly decreased chao 1 (*p* < 0.01) and observed_otus (*p* < 0.01) measures of alpha diversity (Table 8).

**Table 8:** Alpha diversity indexes of cecal microbiota.

Items	CON	CHM	*p*-value
Chao 1	428.16 ± 48.69	307.68 ± 33.63	0.01
Shannon	4.85 ± 0.41	4.44 ± 0.28	0.11
Simpson	0.84 ± 0.06	0.87 ± 0.03	0.32
Goods_coverage	1.00	1.00	-
Pielou_e	0.55 ± 0.04	0.54 ± 0.03	0.45
Observed_otus	428.00 ± 48.56	307.60 ± 33.55	0.01

CON, control; CHM, Chinese herbal medicine.

Figures 1C–E displays the distribution of the cecal microbiota at the phylum and genus levels. *Firmicutes* and *Bacteroidetes* were the most prevalent phyla in the two groups (Figure 1C). CHM significantly increased the relative abundance of Bacteroidetes (*p* = 0.01; Figure 1E) and decreased *Firmicutes* compared to those in the CON group (*p* < 0.01; Figure 1E). Similarly, at the genus level, CHM had a significant effect on the abundance of *Bacteroidales_unclassified* (*p* = 0.01; Figure 1E) and caused a significant decrease in the abundance of *Alistipes* (*p* = 0.01; Figure 1E).

Based on the effect size measurements (LEfSe), Figure 1F shows the identification of 19 biomarkers with linear discriminant analysis (LDA) values exceeding four. In addition, the CHM group exhibited enrichment of six bacterial taxa, namely unidentified *Bacteroidales* (species), unidentified *Bacteroidales* (family), unidentified *Bacteroidales* (genus), *Bacteroidota* (phylum), *Bacteroidales* (order), and *Bacteroidia* (class). Enrichment of the CON group was observed in various taxa including *Rikenellaceae* (family), *Alistipes* (genus), *Alistipes_unclassified* (species), *Clostridia* (class), *Alistipes_ihumii* (species), *Firmicutes* (phylum), *Oscillospirales* (order), *Incertae_sedis_unclassified* (species), *Incertae_sedis* (species), *Clostridiales* (order), *Bacteroides* (genus), *Bacteroides_sp__S461* (species), and *Bacteroidaceae* (family).

### 3.7 Correlation analysis of cecal microbiota, FCR and jejunal mucosal gene changes

**Figure 2:**
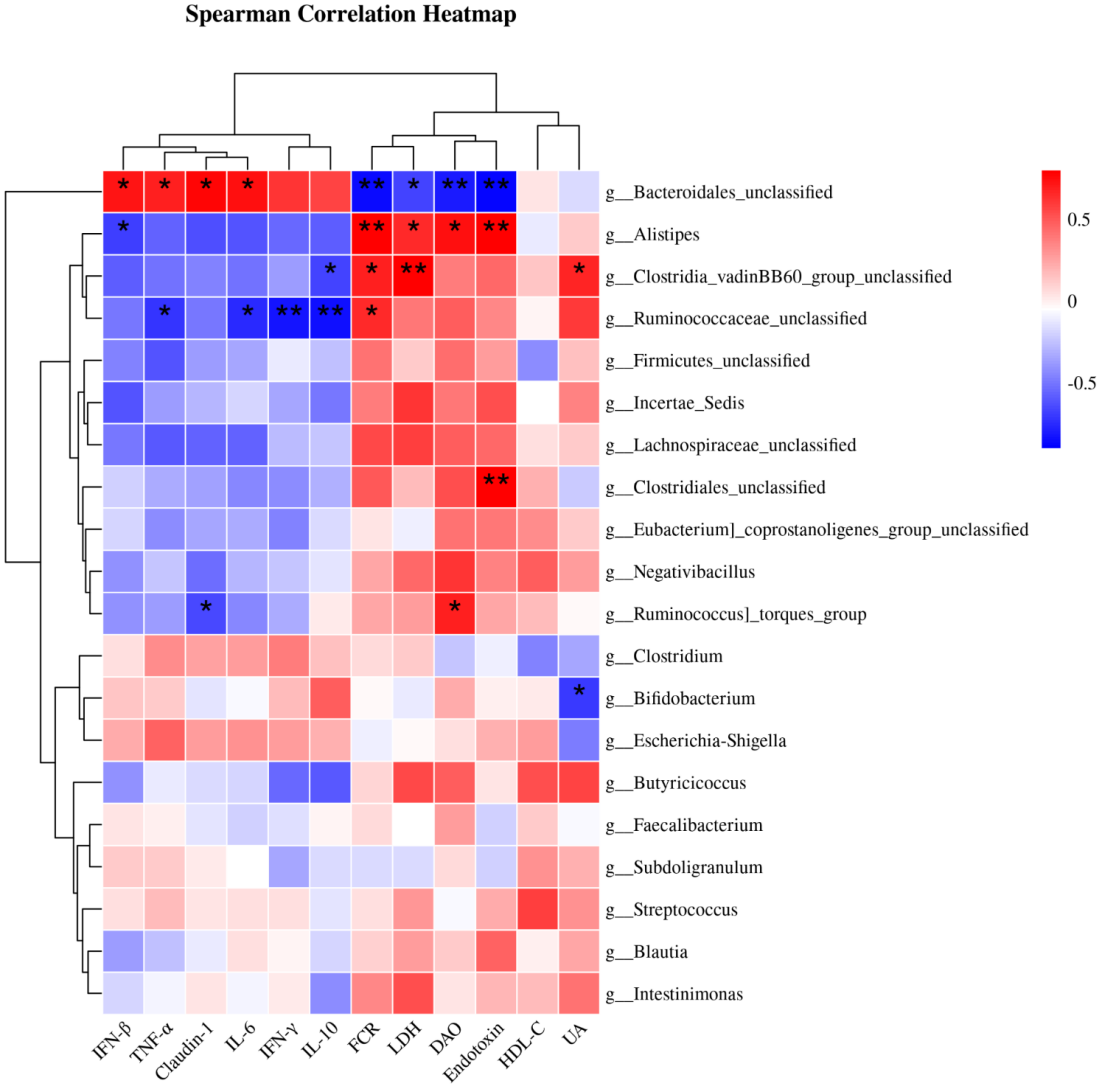
Spearman correlation analysis of cecal microbiota and FCR or serum differential metabolites or genes with significant differences in jejunal mucosa. Statistically significant associations (*p* < 0.05) were observed between certain indicators and bacterial genera. The red blocks indicate positive correlations, while the blue blocks indicate negative correlations. The intensity of the colors reflects the strength of the correlations. * *p* < 0.05 and ** *p* < 0.01 indicate significant differences compared to the CON group. IFN-β, interferon-β; TNF-α, tumor necrosis factor-α; IL-6, interleukin-6; IFN-γ, interferon-γ; IL-10, interleukin-10; FCR, feed conversion ratio; LDH, lactate dehydrogenase; DAO, diamine oxidase; HDL-C, high-density lipoprotein cholesterol; UA, uric acid; CON, control; CHM, Chinese herbal medicine.

Figure 2 displays the results of Spearman’s correlation analysis, illustrating the relationship between the most prominent 20 genus and factors such as FCR, serum differential metabolites (DAO, HDL-C, LDH, UA, and endotoxin), and genes with significant differences in the jejunal mucosa. The abundance of *Bacteroidales_unclassified* had a positive correlation with the expression of IFN-β, TNF-α, Claudin-1, and IL-6 mRNAs, while it had a negative correlation with FCR, DAO, LDH, and endotoxin. However, *Alistipes* showed a negative correlation with the mRNA expression of IFN-β, and a positive correlation with the FCR, DAO, LDH, and endotoxin. Meanwhile, *Ruminococcaceae_unclassified* exhibited a negative correlation with the mRNA expression of IFN-γ, TNF-α, IL-6, and IL-10, while showing a positive correlation with FCR. Furthermore, *Clostridia_vadinBB60_group_unclassified* showed a positive correlation with FCR, LDH and UA and a negative correlation with IL-10 expression.

## 4 Discussion

FCR is used in animal production as a key indicator for evaluating feed utilization efficiency and production benefits. It can be used to assess chicken performance and is frequently used in meat-producing poultry (24). Birds with low FCR are considered as having high feed efficiency. FCR is affected by genetics, health, food, and the environment in which the chickens are raised (24–26). Neither of the two groups of our study differed significantly in BW, ADG, or ADFI; however, the BW and ADG were higher in the CHM group than that in the CON group. Therefore, supplementation with CHM reduced the FCR and improved the feed utilization efficiency. According to previous studies, the injection of AMT components into poultry muscle significantly alleviated the decrease in feed efficiency of lipopolysaccharide (LPS)-treated broilers (27). Other studies have shown that the addition of AMT polysaccharide and Glycyrrhiza uralensis polysaccharide reduced the FCR and increased ADG of broilers (28); however, the ADG between the two groups was not significantly different in our study. We speculate that AMT and CPO extracts contain numerous beneficial components, including polysaccharides, saponins and so on, which stimulate livestock digestion and absorption, enhance nutrient utilization, and reduce feed waste, thus increasing feed efficiency (27, 29–31).

Serum biochemical indexes partially reflect metabolism and health state of the body. UA levels in serum reflect both the production and excretion of UA. The present study indicated that CHM increased the level of HDL-C and reduced those of UA, LDH, DAO, and endotoxin in serum. LDH levels are primarily associated with the extent of cell necrosis and damage to the cell membrane (32). Higher levels of LDH indicate a greater degree of cell necrosis and damage. This increase in LDH levels can be attributed to the anti-inflammatory and antioxidant properties of the extract, as well as its role in regulating cell function. In addition, a positive correlation has been found between endotoxin levels and DAO activity in blood (33). Increased intestinal permeability can compromise intestinal barrier integrity, allowing bacteria and other harmful substances to enter the bloodstream more easily, thereby triggering inflammation and other health issues. Therefore, the addition of CHM can have a positive effect on body metabolism and intestinal barrier function by increasing HDL-C levels and decreasing endotoxin and LDH levels and DAO activity in the blood.

The antioxidant capacity of the body inhibits the production of free radicals and prevents chain reactions of free radicals, thus alleviating oxidative damage to the host. This improvement in antioxidant capacity is beneficial for the health and production performance of broilers (34). T-AOC comprehensively reflects the antioxidant capacity of both enzyme and non-enzymes defense systems (35). Antioxidative enzymes are composed of significant elements such as T-SOD, CAT, and GSH-Px, which have a critical function in eradicating superoxide anions and hydrogen peroxide. These enzymes protect cells and tissues against the harmful effects of oxidative stress (35–37). Wang, et al. (38) reported that diets supplemented with AMT increased sheep CAT, T-SOD, and T-AOC levels in the small intestinal mucosa and meat tissue, improving their antioxidant capacity. Similarly, adding AMT to quails increased their T-AOC, GSH-Px, and CAT activities (39). Our results showed increased serum and liver T-AOC, SOD, and GSH-Px, and liver CAT levels in the CHM group. This increase may be due to the bioactive compounds present in the CPO and AMT extracts mixtures, including polysaccharides, saponins, and flavonoids, which demonstrate significant antioxidant effects (40). Furthermore, research has revealed that these herbs can modulate the immune system, stimulate cell regeneration and repair, and support normal metabolism and physiological functions, while simultaneously counteracting free radical damage within the body (41–43). These effects may be because some compounds in CHM are antioxidants that fight free-radical damage in the body and boost antioxidant activity.

An intact intestinal morphology and a healthy intestinal epithelial barrier are important for maintaining animal health, improving immunity, protecting the host from pathogens, and supporting subsequent growth (44–46). The jejunum serves as the primary site for nutrient absorption, featuring a multitude of villi on the mucosa. The intestinal mucosa has a direct effect on the ability of the host to resist potentially invading pathogens. As villus height increases, both the intestinal surface area and the number of epithelial cells increase, resulting in an enhanced capacity for nutrient absorption (47). In broilers studies, AMT polysaccharide has been shown to increase villus height in the small intestine (48). Our results showed that CHM supplementation increased villus height and the mRNA level of Claudin-1 in the jejunal mucosa. Claudin-1 is an important intestinal tight junction protein that facilitates formation and reinforcement of intercellular junctions, resulting in improved cell epithelial barrier strength and stability (49, 50). These functions were further confirmed by the decreased levels of DAO and endotoxin in the CHM group. The mucosal immune system is an essential component of the immune system (51). In addition, cells of the innate immune system produce important cytokines that contribute to adaptive immunity (52). IL-6 and TNF-α are innate immunity-related cytokines with pro-inflammatory properties that are crucial for host defense, inducing inflammation and triggering apoptosis (53). In contrast, IL-10 is a vital anti-inflammatory cytokine. Elevated levels of IL-10 during inflammation help modulate and balance the inflammatory process, thereby maintaining homeostasis (54). A study demonstrated that CPO induced increased cytokine (IL-2 and IFN-γ) levels, upregulated the expression of the appropriate mRNA in mice, and improved the immune organ index (55). Another study reported that a polysaccharide derived from CPO could potentially have immunomodulatory effects. It was found to enhance the secretion of cytokines (IL-6 and TNF-α) in RAW 264.7 macrophages, without exhibiting any cytotoxic effects (56). Moreover, CPO has been implicated in promoting the production of IL-2, TNF, and IFN in mice upon extraction of pectic polysaccharide (57). Consistent with our present results, we found that the mRNA levels of cytokines IL-6, IL-10, IFN-β, IFN-γ, and TNF-α were elevated in the jejunal mucosa. Therefore, it can be speculated that herbal medicine’s active ingredients act as immunostimulants, activating immune cells and promoting proinflammatory mediator secretion, potentially leading to upregulation of cytokines such as IL-6 and TNF-α, which may subsequently upregulate IFN-β and IFN-γ. Concomitantly, upregulation of the anti-inflammatory cytokine IL-10 maintains homeostasis by counteracting the inflammatory process. These findings indicate that CHM may enhance the gut barrier function and promote intestinal health in broilers.

The gut microflora is crucial for regulating intestinal motility, immune homeostasis, and nutrient absorption (58). Furthermore, research has shown that intestinal flora can assist the host in protecting against pathogens and inflammatory bowel diseases, thus enhancing the host’s digestive system health (59). Chao 1 is a measure of the community richness. Our findings showed that the addition of CHM reduced microbial richness and the observed number of OUT’s, which was different from other studies (28, 60). This may be associated with the ability of CHM to eliminate and impede pathogenic disease-causing microbes in the gut. Furthermore, NMDS analysis indicated that the microbial communities in the CON and CHM groups differed in composition. At the phylum level, our results indicated that *Firmicutes* and *Bacteroides* dominated the cecal microflora, which was consistent with the results of previous studies (61, 62). The ratio of *Firmicutes* to *Bacteroidetes* is an important indicator of gut microbiota health (63), whereas inflammation and gut permeability improve when the ratio of *Firmicutes* to *Bacteroides* decreases (64). Studies have suggested that the addition of herbal medicines, such as CPO and AMT, decreases the ratio of *Firmicutes* to *Bacteroidetes* in weaned piglets (17), which is consistent with our results. There is evidence that *Bacteroidetes* and *Firmicutes* contribute to broiler digestion (65), and *Bacteroides* genus displays a positive impact on host health and disease resistance (66). In addition, we observed a higher abundance of *Bacteroidales_unclassified* and lower abundance of *Alistipes* after CHM supplementation. A study previous found that inhibiting the proportion of *Alistipes* increased its metabolism (67). In addition, *Alistipes*, a microbe that produces indole, can disrupt the serotonin balance in the gut through excessive growth (68). Hence, adding AMT and CPO extracts to broiler diets could enhance gut microbiota and health by increasing *Bacteroidetes* abundance and modulating the *Firmicutes* to *Bacteroides* ratio.

Spearman’s correlation analysis revealed that the cecum microbiota correlated with the FCR, serum differential metabolites, and gene expression in the jejunal mucosa. *Bacteroides* are frequently associated with the decomposition of polysaccharides, particularly starch and glucan (69). We found that *Bacteroidales_unclassified* was positively correlated with immune factors and tight junction protein mRNA expression, whereas it was negatively correlated with the FCR. Similar with our results, previous studies have found a positive relationship between the *Bacteroidetes* phylum and the plasma level of the pro-inflammatory cytokine TNF-α (70). The genus *Bacteroides* was linked to the cytokine IL-6 produced by monocytes and maintained the integrity of the epithelial barrier by controlling intraepithelial lymphocytes (from which IL-6 is formed) (71, 72). *Bacteroides* spp. have been proven to induce macrophages and monocytes to release TNF-α through LPS-mediated pathways (73). It has also been reported that *Bacteroides fragilis* supplementation enhances the expression of the tightly wound response proteins Claudin-1 using real-time qPCR and immunofluorescence staining (74). In contrast to the genus *Bacteroidales_unclassified* in the correlation analysis, we found that the genus *Alistipes* showed a negative correlation with the mRNA expression of immune factors and tight junction protein factor mRNA and a positive correlation with the FCR, which was different from the findings of previous studies (75, 76). Given that *Alistipes* is a subbranch of *Bacteroidetes* that is relatively recent in terms of pathogenicity, comparative data suggest that *Alistipes* may cause colon cancer (77). Moreover, *Alistipes*, a possible pathogen, is implicated in the pathogenesis, which thrives in an inflammatory environment devoid of lipocalin 2, encouraging inflammation and tumor development (78). The present study showed that CHM supplementation increased *Bacteroidales_unclassified* abundance and decreased *Alistipes* abundance. Therefore, CHM supplementation improved feed efficiency and increased the mRNA expression of pro-inflammatory cytokines in the jejunal mucosa. Additionally, CHM supplementation decreased the level of endotoxin and activities of DAO and LDH in the serum. These effects may be associated with alterations in the broiler gut microbiota.

## 5 Conclusion

Dietary supplementation with 500 mg/kg CHM (AMT and CPO extracts) improved the FCR and antioxidant capacity in the serum and liver and decreased the levels of UA and endotoxin and activity of DAO. Moreover, dietary CHM raised the concentrations of IL-6, IFN-γ, IFN-β, TNF-α, and the anti-inflammatory cytokine IL-10, while it induced alterations in the microbial composition of the cecum in broiler chickens. Spearman correlation analysis identified a correlation between cecal microbiota composition and FCR or serum differential metabolites or genes with significant differences in the jejunal mucosa, which might be attributed to the increased *Bacteroidales_unclassified* abundance and decreased *Alistipes* abundance in broilers. Further research is needed to explore the effects and mechanisms of the active ingredients in CHM, primarily AMT and CPO extracts, on animal health.

## Data availability statement

The original contributions presented in the study are publicly available. This data can be found here: https://www.ncbi.nlm.nih.gov/bioproject/; PRJNA1023878.

## Ethics statement

The animal study was approved by Animal Care and Use Committee of Foshan University (approval ID: FOSU#19-025). The study was conducted in accordance with the local legislation and institutional requirements.

## Author contributions

SL: Data curation, Methodology, Writing – original draft, Writing – review & editing. GX: Data curation, Methodology, Writing – original draft. QW: Investigation, Software, Writing – original draft. JT: Investigation, Software, Writing – original draft. XF: Writing – review & editing. QZ: Investigation, Writing – original draft. LG: Conceptualization, Funding acquisition, Supervision, Writing – review & editing.

## Footnotes

^1^ http://www.njjcbio.com/

^2^ https://www.omicstudio.cn/tool/60

^3^ https://www.omicstudio.cn

^4^ https://www.omicstudio.cn/tool

## References

[1] Cheng K Song ZH Zheng XC Zhang H Zhang JF Zhang LL. Effects of dietary vitamin E type on the growth performance and antioxidant capacity in cyclophosphamide immunosuppressed broilers. Poult Sci. (2017) 96:1159–66. doi: 10.3382/ps/pew336, PMID: 27665006

[2] Gao J Yang Z Zhao C Tang X Jiang Q Yin Y. A comprehensive review on natural phenolic compounds as alternatives to in-feed antibiotics. Sci China Life Sci. (2022) 66:1518–34. doi: 10.1007/s11427-022-2246-4, PMID: 36586071

[3] Ma HD Deng YR Tian Z Lian ZX. Traditional Chinese medicine and immune regulation. Clin Rev Allergy Immunol. (2013) 44:229–41. doi: 10.1007/s12016-012-8332-0 22826112

[4] Costa LB Luciano FB Miyada VS Gois FD. Herbal extracts and organic acids as natural feed additives in pig diets. S Afr J Anim Sci. (2013) 43:181–93. doi: 10.4314/sajas.v43i2.9

[5] Zhao L Zhao JL Bai Z Du J Shi Y Wang Y. Polysaccharide from dandelion enriched nutritional composition, antioxidant capacity, and inhibited bioaccumulation and inflammation in *Channa asiatica* under hexavalent chromium exposure. Int J Biol Macromol. (2022) 201:557–68. doi: 10.1016/j.ijbiomac.2021.12.117, PMID: 35007636

[6] Simeonova R Bratkov VM Kondeva-Burdina M Vitcheva V Manov V Krasteva I. Experimental liver protection of n-butanolic extract of *Astragalus monspessulanus* L. on carbon tetrachloride model of toxicity in rat. Redox Rep. (2015) 20:145–53. doi: 10.1179/1351000214Y.0000000115, PMID: 25396696 PMC6837432

[7] Yang LP Shen JG Xu WC Li J Jiang JQ. Secondary metabolites of the genus Astragalus: structure and biological-activity update. Chem Biodivers. (2013) 10:1004–54. doi: 10.1002/cbdv.201100444, PMID: 23776019

[8] Guo Z Lou Y Kong M Luo Q Liu Z Wu J. A systematic review of Phytochemistry, pharmacology and pharmacokinetics on Astragali Radix: implications for Astragali Radix as a personalized medicine. Int J Mol Sci. (2019) 20:1463. doi: 10.3390/ijms20061463 30909474 PMC6470777

[9] Chang CD Yi YX. Protective effect of rhubarb on barrier of intestinal mucosa. Chinese Crit Care Med. (1997) 3:81–3.

[10] Zhao G Dai S Chen R. Dictionary of traditional Chinese medicine. Beijing: Commercial Press (2006).

[11] Ng TB Liu F Wang HX. The antioxidant effects of aqueous and organic extracts of Panax quinquefolium, Panax notoginseng, *Codonopsis pilosula*, Pseudostellaria heterophylla and *Glehnia littoralis*. J Ethnopharmacol. (2004) 93:285–8. doi: 10.1016/j.jep.2004.03.040, PMID: 15234766

[12] Singh B Song H Liu XD Hardy M Liu GZ Vinjamury SP. Dangshen (*Codonopsis pilosula*) and Bai guo (Gingko biloba) enhance learning and memory. Altern Ther Health Med. (2004) 10:52–6. doi: 10.1016/j.jep.2004.05.001 PMID: 15285274

[13] Zhao X Hu Y Wang D Liu J Guo L. The comparison of immune-enhancing activity of sulfated polysaccharidses from Tremella and Condonpsis pilosula. Carbohydr Polym. (2013) 98:438–43. doi: 10.1016/j.carbpol.2013.06.043, PMID: 23987365

[14] Weidong Z Lei X Hanqi LU Zewei C Qiangfeng G Ren L. Radix Codonopsi polysaccharide against 5-fluorouracil-induced gastrointestinal mucositis in mice model. Liaoning. J Tradit Chin Med. (2016) 43:1495–8. doi: 10.13192/j.issn.1000-1719.2016.07.051

[15] Gao S Liu J Wang M Liu Y Meng X Zhang T. Exploring on the bioactive markers of Codonopsis Radix by correlation analysis between chemical constituents and pharmacological effects. J Ethnopharmacol. (2019) 236:31–41. doi: 10.1016/j.jep.2019.02.032, PMID: 30776470

[16] Tang S Liu W Zhao Q Li K Zhu J Yao W. Combination of polysaccharides from Astragalus membranaceus and *Codonopsis pilosula* ameliorated mice colitis and underlying mechanisms. J Ethnopharmacol. (2021) 264:113280. doi: 10.1016/j.jep.2020.113280, PMID: 32822821

[17] Wang M Huang H Wang L Yang H He S Liu F. Herbal extract mixture modulates intestinal Antioxidative capacity and microbiota in weaning piglets. Front Microbiol. (2021) 12:706758. doi: 10.3389/fmicb.2021.706758, PMID: 34394056 PMC8357371

[18] Xiao G Liu S Yan X Yang Y Qi Q Feng X. Effects of fulvic acid addition on laying performance, biochemical indices, and gut microbiota of aged hens. Front Vet Sci. (2022) 9:953564. doi: 10.3389/fvets.2022.953564, PMID: 36118354 PMC9479332

[19] Cui J Hao Z Zhou Q Qiu M Liu Y Liu Y. Chlorpyrifos induced autophagy and mitophagy in common carp livers through AMPK pathway activated by energy metabolism disorder. Ecotoxicol Environ Saf. (2023) 258:114983. doi: 10.1016/j.ecoenv.2023.114983 37148751

[20] Liu Y Lin X Hao Z Yu M Tang Y Teng X. Cadmium exposure caused cardiotoxicity in common carps (*Cyprinus carpio* L.): miR-9-5p, oxidative stress, energetic impairment, mitochondrial division/fusion imbalance, inflammation, and autophagy. Fish Shellfish Immunol. (2023) 138:108853. doi: 10.1016/j.fsi.2023.108853, PMID: 37245677

[21] Hashemi SM Loh TC Foo HL Zulkifli I Hair-Bejo M. Dietary putrescine effects on performance parameters, nutrient digestibility, intestinal morphology and tissue polyamine content of broilers fed low protein diet. Iran J Vet Res. (2014) 15:385–91. doi: 10.22099/IJVR.2014.2597 PMID: 27175136 PMC4789218

[22] Cui J Liu Y Hao Z Liu Y Qiu M Kang L. Cadmium induced time-dependent kidney injury in common carp via mitochondrial pathway: impaired mitochondrial energy metabolism and mitochondrion-dependent apoptosis. Aquat Toxicol. (2023) 261:106570. doi: 10.1016/j.aquatox.2023.106570, PMID: 37202229

[23] Best DJ Roberts DE. The upper tail probabilities of Spearman's rho. Appl Stat. (1975) 24:377. doi: 10.2307/2347111

[24] Aggrey SE Karnuah AB Sebastian B Anthony NB. Genetic properties of feed efficiency parameters in meat-type chickens. Genet Sel Evol. (2010) 42:25. doi: 10.1186/1297-9686-42-25, PMID: 20584334 PMC2901204

[25] Awad WA Ghareeb K Abdel-Raheem S Böhm J. Effects of dietary inclusion of probiotic and synbiotic on growth performance, organ weights, and intestinal histomorphology of broiler chickens. Poult Sci. (2009) 88:49–56. doi: 10.3382/ps.2008-00244, PMID: 19096056

[26] Pedroso AA Menten JF Lambais MR Racanicci AM Longo FA Sorbara JO. Intestinal bacterial community and growth performance of chickens fed diets containing antibiotics. Poult Sci. (2006) 85:747–52. doi: 10.1093/ps/85.4.747 16615359

[27] Wang X Li Y Shen J Wang S Yao J Yang X. Effect of Astragalus polysaccharide and its sulfated derivative on growth performance and immune condition of lipopolysaccharide-treated broilers. Int J Biol Macromol. (2015) 76:188–94. doi: 10.1016/j.ijbiomac.2015.02.040, PMID: 25748840

[28] Qiao Y Liu C Guo Y Zhang W Guo W Oleksandr K. Polysaccharides derived from Astragalus membranaceus and Glycyrrhiza uralensis improve growth performance of broilers by enhancing intestinal health and modulating gut microbiota. Poult Sci. (2022) 101:101905. doi: 10.1016/j.psj.2022.101905, PMID: 35576745 PMC9117935

[29] Tan C Leng X Li X Su X Liu B Chai X. Effects of polysaccharides, oligosaccharides and protease on growth, digestive enzyme activities and serum nonspecific immunity of white shrimp (*Litopenaeus vannamei*). J Shanghai Ocean University. (2013) 22:93–9.

[30] Wu S. Effect of dietary Astragalus membranaceus polysaccharide on the growth performance and immunity of juvenile broilers. Poult Sci. (2018) 97:3489–93. doi: 10.3382/ps/pey220, PMID: 29897509

[31] Luan F Ji Y Peng L Liu Q Cao H Yang Y. Extraction, purification, structural characteristics and biological properties of the polysaccharides from *Codonopsis pilosula*: a review. Carbohydr Polym. (2021) 261:117863. doi: 10.1016/j.carbpol.2021.117863, PMID: 33766352

[32] Zhuang X Jin K Li X Li J. Autoimmune glial fibrillary acidic protein astrocytopathy in children: a retrospective study. Eur J Med Res. (2022) 27:11. doi: 10.1186/s40001-022-00641-y, PMID: 35065659 PMC8783492

[33] Cheng C Wei H Xu C Xie X Jiang S Peng J. Maternal soluble Fiber diet during pregnancy changes the intestinal microbiota, improves growth performance, and reduces intestinal permeability in piglets. Appl Environ Microbiol. (2018) 84:e01047-18. doi: 10.1128/AEM.01047-18, PMID: 29959248 PMC6102992

[34] Yang Z Liu C Zheng W Teng X Li S. The functions of antioxidants and heat shock proteins are altered in the immune organs of selenium-deficient broiler chickens. Biol Trace Elem Res. (2016) 169:341–51. doi: 10.1007/s12011-015-0407-3, PMID: 26123162

[35] Birben E Sahiner UM Sackesen C Erzurum S Kalayci O. Oxidative stress and antioxidant defense. World Allergy Organ J. (2012) 5:9–19. doi: 10.1097/WOX.0b013e3182439613, PMID: 23268465 PMC3488923

[36] Fukai T Ushio-Fukai M. Superoxide dismutases: role in redox signaling, vascular function, and diseases. Antioxid Redox Signal. (2011) 15:1583–606. doi: 10.1089/ars.2011.3999, PMID: 21473702 PMC3151424

[37] Shang X Xu W Zhang Y Sun Q Li Z Geng L. Transcriptome analysis revealed the mechanism of Luciobarbus capito (*L. capito*) adapting high salinity: antioxidant capacity, heat shock proteins, immunity. Mar Pollut Bull. (2023) 192:115017. doi: 10.1016/j.marpolbul.2023.115017 37172343

[38] Wang X Hu C Ding L Tang Y Wei H Jiang C. Astragalus membranaceus alters rumen Bacteria to enhance Fiber digestion, improves antioxidant capacity and immunity indices of small intestinal mucosa, and enhances liver metabolites for energy synthesis in Tibetan sheep. Animals. (2021) 11:3236. doi: 10.3390/ani11113236 34827968 PMC8614378

[39] Guo L Hua J Luan Z Xue P Zhou S Wang X. Effects of the stems and leaves of Astragalus membranaceus on growth performance, immunological parameters, antioxidant status, and intestinal bacteria of quail. Anim Sci J. (2019) 90:747–56. doi: 10.1111/asj.13213, PMID: 30989748

[40] Yu QT Qi LW Li P Yi L Zhao J Bi ZM. Determination of seventeen main flavonoids and saponins in the medicinal plant Huang-qi (Radix Astragali) by HPLC-DAD-ELSD. J Sep Sci. (2007) 30:1292–9. doi: 10.1002/jssc.200600422, PMID: 17623470

[41] Shen X Zhao Z Wang H Guo Z Hu B Zhang G. Elucidation of the anti-inflammatory mechanisms of Bupleuri and Scutellariae Radix using system pharmacological analyses. Mediat Inflamm. (2017) 2017:1–10. doi: 10.1155/2017/3709874 PMC5278517 28190938

[42] Ge X Yao T Zhang C Wang Q Wang X Xu LC. Human microRNA-4433 (hsa-miR-4443) targets 18 genes to be a risk factor of neurodegenerative diseases. Curr Alzheimer Res. (2022) 19:511–22. doi: 10.2174/1567205019666220805120303, PMID: 35929619 PMC9906632

[43] Xu R Lin L Li Y Li Y. ShenQi FuZheng injection combined with chemotherapy in the treatment of colorectal cancer: a meta-analysis. PLoS One. (2017) 12:e0185254. doi: 10.1371/journal.pone.0185254, PMID: 28953950 PMC5617195

[44] Prakatur I Miskulin M Pavic M Marjanovic K Blazicevic V Miskulin I. Intestinal morphology in broiler chickens supplemented with propolis and bee pollen. Animals. (2019) 9:301. doi: 10.3390/ani9060301 31151310 PMC6617278

[45] Chen Y-W Yu Y-H. Differential effects of *Bacillus subtilis*– and *Bacillus licheniformis*–fermented products on growth performance, intestinal morphology, intestinal antioxidant and barrier function gene expression, cecal microbiota community, and microbial carbohydrate-active enzyme composition in broilers. Poult Sci. (2023) 102:102670. doi: 10.1016/j.psj.2023.102670 37068351 PMC10130491

[46] Shi X Xu W Che X Cui J Shang X Teng X. Effect of arsenic stress on the intestinal structural integrity and intestinal flora abundance of *Cyprinus carpio*. Front Microbiol. (2023) 14:1179397. doi: 10.3389/fmicb.2023.1179397, PMID: 37168116 PMC10165157

[47] Shan C Sun B Dalloul RA Zhai Z Sun P Li M. Effect of the oral administration of astragalus polysaccharides on jejunum mucosal immunity in chickens vaccinated against Newcastle disease. Microb Pathog. (2019) 135:103621. doi: 10.1016/j.micpath.2019.103621, PMID: 31310831

[48] Yang S-b Qin Y-j Ma X Luan W-m Sun P Ju A-q. Effects of in ovo injection of Astragalus polysaccharide on the intestinal development and mucosal immunity in broiler chickens. Frontiers in veterinary. Science. (2021) 8:738816. doi: 10.3389/fvets.2021.738816 PMC8435677 34527718

[49] Pope JL Bhat AA Sharma A Ahmad R Krishnan M Washington MK. Claudin-1 regulates intestinal epithelial homeostasis through the modulation of notch-signalling. Gut. (2014) 63:622–34. doi: 10.1136/gutjnl-2012-304241, PMID: 23766441 PMC4083824

[50] Wang R Yang X Liu J Zhong F Zhang C Chen Y. Gut microbiota regulates acute myeloid leukaemia via alteration of intestinal barrier function mediated by butyrate. Nat Commun. (2022) 13:2522. doi: 10.1038/s41467-022-30240-8, PMID: 35534496 PMC9085760

[51] Gallego M del Cacho E Bascuas JA. Antigen-binding cells in the cecal tonsil and Peyer's patches of the chicken after bovine serum albumin administration. Poult Sci. (1995) 74:472–9. doi: 10.3382/ps.0740472, PMID: 7761331

[52] Jeurissen SH Lewis F van der Klis JD Mroz Z Rebel JM ter Huurne AA. Parameters and techniques to determine intestinal health of poultry as constituted by immunity, integrity, and functionality. Curr Issues Intest Microbiol. (2002) 3:1–14. PMID: 12022808

[53] Al-Banna NA Cyprian F Albert MJ. Cytokine responses in campylobacteriosis: linking pathogenesis to immunity. Cytokine Growth Factor Rev. (2018) 41:75–87. doi: 10.1016/j.cytogfr.2018.03.005, PMID: 29550265

[54] Fukuhara YD Reis ML Dellalibera-Joviliano R Cunha FQ Donadi EA. Increased plasma levels of IL-1beta, IL-6, IL-8, IL-10 and TNF-alpha in patients moderately or severely envenomed by Tityus serrulatus scorpion sting. Toxicon. (2003) 41:49–55. doi: 10.1016/S0041-0101(02)00208-8 12467661

[55] Bai R-B Zhang Y-J Fan J-M Jia X-S Li D Wang Y-P. Immune-enhancement effects of oligosaccharides from *Codonopsis pilosula* on cyclophosphamide induced immunosuppression in mice. Food Funct. (2020) 11:3306–15. doi: 10.1039/C9FO02969A 32227014

[56] Ji H-Y Yu J Jiao J-S Dong X-D Yu S-S Liu A-J. Ultrasonic-assisted extraction of *Codonopsis pilosula* Glucofructan: optimization, structure, and immunoregulatory activity. Nutrients. (2022) 14:927. doi: 10.3390/nu14050927, PMID: 35267905 PMC8912531

[57] Zhang P Hu L Bai R Zheng X Ma Y Gao X. Structural characterization of a pectic polysaccharide from Codonopsis pilosula and its immunomodulatory activities in vivo and in vitro. Int J Biol Macromol. (2017) 104:1359–69. doi: 10.1016/j.ijbiomac.2017.06.023, PMID: 28600205

[58] Kau AL Ahern PP Griffin NW Goodman AL Gordon JI. Human nutrition, the gut microbiome and the immune system. Nature. (2011) 474:327–36. doi: 10.1038/nature10213, PMID: 21677749 PMC3298082

[59] Li JT Tao LJ Zhang R Yang GQ. Effects of fermented feed on growth performance, nutrient metabolism and cecal microflora of broilers. Anim Biosci. (2022) 35:596–604. doi: 10.5713/ab.21.0333, PMID: 34727643 PMC8902206

[60] Che D Adams S Wei C Gui-Xin Q Atiba EM Hailong J. Effects of Astragalus membranaceus fiber on growth performance, nutrient digestibility, microbial composition, VFA production, gut pH, and immunity of weaned pigs. Microbiologyopen. (2018) 8:e00712. doi: 10.1002/mbo3.712 30117299 PMC6528644

[61] Waite DW Taylor MW. Characterizing the avian gut microbiota: membership, driving influences, and potential function. Front Microbiol. (2014) 5:223. doi: 10.3389/fmicb.2014.00223 24904538 PMC4032936

[62] Stanley D Geier MS Denman SE Haring VR Crowley TM Hughes RJ. Identification of chicken intestinal microbiota correlated with the efficiency of energy extraction from feed. Vet Microbiol. (2013) 164:85–92. doi: 10.1016/j.vetmic.2013.01.030, PMID: 23434185

[63] Yue W Han F. Effects of monoglucoside and diglucoside anthocyanins from Yan 73 (*Vitis vinifera* L.) and spine grape (Vitis davidii Foex) skin on intestinal microbiota in vitro. Food Chem X. (2022) 16:100501. doi: 10.1016/j.fochx.2022.100501, PMID: 36519088 PMC9743158

[64] Everard A Lazarevic V Derrien M Girard M Muccioli GG Neyrinck AM. Responses of gut microbiota and glucose and lipid metabolism to prebiotics in genetic obese and diet-induced leptin-resistant mice. Diabetes. (2011) 60:2775–86. doi: 10.2337/db11-0227, PMID: 21933985 PMC3198091

[65] Wexler HM. Bacteroides: the good, the bad, and the nitty-gritty. Clin Microbiol Rev. (2007) 20:593–621. doi: 10.1128/CMR.00008-07, PMID: 17934076 PMC2176045

[66] Wang C Xiao Y Yu L Tian F Zhao J Zhang H. Protective effects of different *Bacteroides vulgatus* strains against lipopolysaccharide-induced acute intestinal injury, and their underlying functional genes. J Adv Res. (2022) 36:27–37. doi: 10.1016/j.jare.2021.06.012, PMID: 35127162 PMC8799915

[67] Song X Zhong L Lyu N Liu F Li B Hao Y. Inulin can alleviate metabolism disorders in Ob/Ob mice by partially restoring leptin-related pathways mediated by gut microbiota. Genomics Proteomics Bioinformatics. (2019) 17:64–75. doi: 10.1016/j.gpb.2019.03.001, PMID: 31026583 PMC6520907

[68] Lu WW Fu TX Wang Q Chen YL Li TY Wu GL. The effect of total glucoside of paeony on gut microbiota in NOD mice with Sjögren's syndrome based on high-throughput sequencing of 16SrRNA gene. Chin Med. (2020) 15:61. doi: 10.1186/s13020-020-00342-w, PMID: 32536964 PMC7291443

[69] Beckmann L Simon O Vahjen W. Isolation and identification of mixed linked beta -glucan degrading bacteria in the intestine of broiler chickens and partial characterization of respective 1,3-1,4-beta -glucanase activities. J Basic Microbiol. (2006) 46:175–85. doi: 10.1002/jobm.200510107, PMID: 16721874

[70] Lin CH Chen CC Chiang HL Liou JM Chang CM Lu TP. Altered gut microbiota and inflammatory cytokine responses in patients with Parkinson's disease. J Neuroinflammation. (2019) 16:129. doi: 10.1186/s12974-019-1528-y, PMID: 31248424 PMC6598278

[71] Schirmer M Smeekens SP Vlamakis H Jaeger M Oosting M Franzosa EA. Linking the human gut microbiome to inflammatory cytokine production capacity. Cells. (2016) 167:1125–36.e8. doi: 10.1016/j.cell.2016.10.020, PMID: 27814509 PMC5131922

[72] Kuhn KA Schulz HM Regner EH Severs EL Hendrickson JD Mehta G. Bacteroidales recruit IL-6-producing intraepithelial lymphocytes in the colon to promote barrier integrity. Mucosal Immunol. (2018) 11:357–68. doi: 10.1038/mi.2017.55, PMID: 28812548 PMC5815964

[73] Delahooke DM Barclay GR Poxton IR. Tumor necrosis factor induction by an aqueous phenol-extracted lipopolysaccharide complex from Bacteroides species. Infect Immun. (1995) 63:840–6. doi: 10.1128/iai.63.3.840-846.1995, PMID: 7532627 PMC173079

[74] Wang C Li S Hong K Yu L Tian F Zhao J. The roles of different *Bacteroides fragilis* strains in protecting against DSS-induced ulcerative colitis and related functional genes. Food Funct. (2021) 12:8300–13. doi: 10.1039/D1FO00875G 34308455

[75] Liu Y Yan T Ren Z Yang X. Age-associated changes in caecal microbiome and their apparent correlations with growth performances of layer pullets. Anim Nutr. (2021) 7:841–8. doi: 10.1016/j.aninu.2020.11.019, PMID: 34466688 PMC8379648

[76] Li Y Xu Q Huang Z Lv L Liu X Yin C. Effect of *Bacillus subtilis* CGMCC 1.1086 on the growth performance and intestinal microbiota of broilers. J Appl Microbiol. (2016) 120:195–204. doi: 10.1111/jam.12972 26480894

[77] Parker BJ Wearsch PA Veloo ACM Rodriguez-Palacios A. The genus Alistipes: gut Bacteria with emerging implications to inflammation, Cancer, and mental health. Front Immunol. (2020) 11:906. doi: 10.3389/fimmu.2020.00906, PMID: 32582143 PMC7296073

[78] Goetz DH Holmes MA Borregaard N Bluhm ME Raymond KN Strong RK. The neutrophil lipocalin NGAL is a bacteriostatic agent that interferes with siderophore-mediated iron acquisition. Mol Cell. (2002) 10:1033–43. doi: 10.1016/S1097-2765(02)00708-6, PMID: 12453412

